# High absolute neutrophil count with type 2 diabetes is associated with adverse outcome in patients with coronary artery disease: A large-scale cohort study

**DOI:** 10.3389/fendo.2023.1129633

**Published:** 2023-04-11

**Authors:** Jining He, Zhangyu Lin, Chenxi Song, Rui Zhang, Haoyu Wang, Sheng Yuan, Xiaohui Bian, Qiuting Dong, Kefei Dou

**Affiliations:** ^1^ Cardiometabolic Medicine Center, State Key Laboratory of Cardiovascular Disease, Beijing, China; ^2^ Cardiometabolic Medicine Center, Fuwai Hospital, National Center for Cardiovascular Diseases, Chinese Academy of Medical Sciences and Peking Union Medical College, Beijing, China; ^3^ Department of Cardiology, Fuwai Hospital, National Center for Cardiovascular Diseases, Chinese Academy of Medical Sciences and Peking Union Medical College, Beijing, China

**Keywords:** coronary artery disease, neutrophils, percutaneous coronary intervention, prognosis, type 2 diabetes

## Abstract

**Background:**

Inflammatory processes crucially modulate the development, progression, and outcomes of coronary artery disease (CAD). Since hyperglycemia could alter inflammatory responses, this study aimed to investigate the effect of ANC, a novel and rapidly available inflammatory biomarker, on the prognosis of patients undergoing PCI with or without type 2 diabetes (T2D).

**Methods:**

A total of 7,826 patients with CAD hospitalized for PCI at Fuwai Hospital were consecutively recruited. According to the median ANC value, patients were stratified as having high ANC (ANC-H) or low ANC (ANC-L) and were further classified into four groups by T2D. The primary endpoint was major adverse cardiovascular and cerebrovascular events (MACCEs), including all-cause mortality, myocardial infarction, stroke, and target vessel revascularization.

**Results:**

During a median follow-up of 2.4 years, 509 (6.5%) MACCEs were documented. Diabetic patients with increased ANC were at significantly higher risk of MACCEs (aHR, 1.55; 95% CI, 1.21–1.99; P = 0.001) compared to those in the ANC-L/non-T2D group (P for interaction between T2D and ANC categories = 0.044). Meanwhile, multivariable regression analysis demonstrated the highest MACCE risk in diabetic patients with a higher level of ANC than others (P for trend <0.001).

**Conclusion:**

This study suggests that stratification of patients with elevated ANC and T2D could provide prognostic information for CAD patients undergoing PCI.

## Introduction

Inflammatory processes crucially modulate the development, progression, and outcomes of cardiovascular (CV) diseases (CAD), and accordingly, targeting inflammation is promising to reduce the atherosclerotic burden (1). Currently, several recent clinical trials have illustrated that there are clinical benefits for patients with CVD by targeting inflammatory pathways. The Canakinumab Anti-inflammatory Thrombosis Outcome Study (CANTOS) (2), followed by the Colchicine Cardiovascular Outcomes Trial (COLCOT) (3), and the second Low Dose Colchicine trial (LoDoCo-2) (4), collectively demonstrated the efficacy of anti-inflammatory treatments in the context of CV prevention. Notably, these contemporary studies not only increased interest in traditional inflammatory biomarkers, including interleukin (IL)-1, IL-6, and C-reactive protein (CRP), but also in finding a simple biomarker widely and rapidly available to clinical practice, such as the absolute neutrophil count (ANC) (5).

Neutrophils are the most abundant subtype of white blood cells in the human circulation and the main cell type during acute inflammatory responses. They belong to the first line of innate immunity and contribute to atherosclerosis in a stage-dependent manner (6). In the initial stage of atherosclerosis, neutrophils attract monocytes to plaques by secreting granule proteins and activate plaque endothelial cells through reactive oxygen species and proteases (7–9). During atherosclerotic progression, neutrophil-derived granule proteins promote the activation of plaque macrophages towards a proinflammatory phenotype (10). Further, myeloperoxidase is released by neutrophils, which increases foam cell formation (11). Also, neutrophil extracellular traps (NETs) activate plaque macrophages to secrete IL-1β and IL-18 through the nucleotide-binding domain and leucine-rich repeat protein 3 (NLRP3) inflammasome formation (10, 12). Regarding advanced atherosclerosis, neutrophils release NETs, which contain cytotoxic histone H4 and matrix metalloproteinases, leading to the breakdown of vascular smooth muscle cells and the extracellular matrix, eventually resulting in fibrous cap thinning, a characteristic of vulnerable plaques (6, 13). Previous studies demonstrated that ANC was positively associated with the rate of adverse clinical events (5, 14). Meanwhile, in the placebo group of the CANTOS trial, the ANC remained stable during the follow-up period (5), which also indicated that it could be a promising candidate biomarker to be accepted by the physicians.

Type 2 diabetes (T2D) is an established predictor for coronary artery disease (CAD), which has been previously suggested to be closely associated with a greater burden of atherosclerotic plaque and fuel the risk of adverse clinical outcomes. Cumulative translational evidence suggests a synergism between neutrophils and hyperglycemia (6, 13). However, the association of ANC with poor prognosis in CAD patients with different glycemic states after percutaneous coronary intervention (PCI) remains uncertain. Therefore, the aim of this study is to investigate the effect of ANC on the clinical outcomes of patients undergoing PCI with or without T2D.

## Methods

### Study design

**Figure 1:**
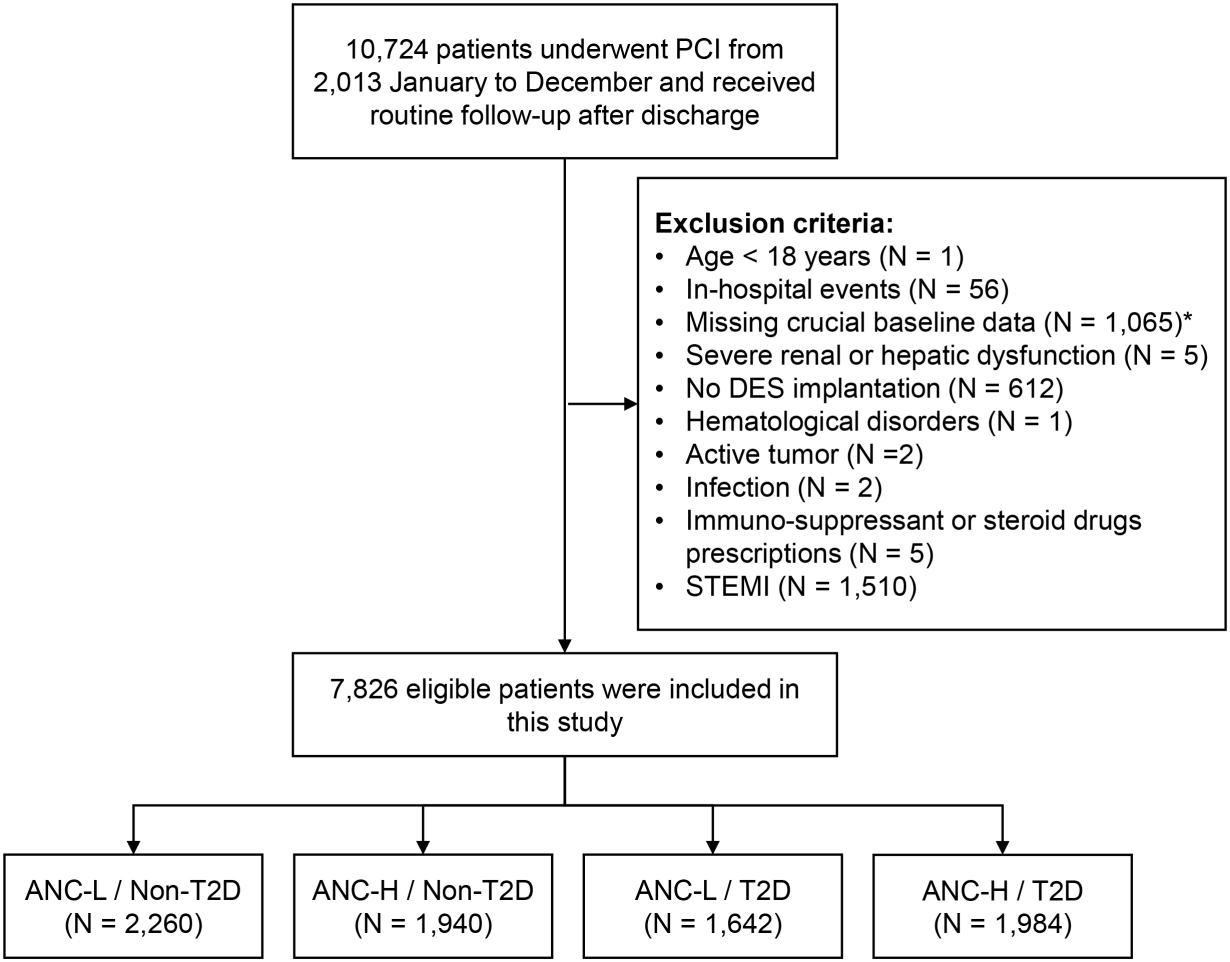
Study flowchart. *A total of 708 patients with missing ANC and 409 patients with missing FBG or HbA1c levels were excluded. PCI, percutaneous coronary intervention; DES, drug-eluting stent; STEMI, ST-segment-elevation myocardial infarction; ANC, absolute neutrophil counts; T2D, type 2 diabetes; FBG, fasting blood glucose; HbA1c, glycosylated hemoglobin A1c.

This study was a prospective, observational cohort study at Fuwai Hospital, Chinese Academy of Medical Sciences. Between January 2013 and December 2013, a total of 10,724 consecutive CAD patients hospitalized for PCI with drug-eluting stent (DES) implantation were screened. The exclusion criteria included (1): patients aged <18 years old; (2) patients with in-hospital death; (3) patients without ANC, fasting blood glucose (FBG), and glycosylated hemoglobin A1c (HbA1c) values; (4) patients with severe kidney or liver dysfunction; (5) patients with hematological disorders, active tumors, acute infections, or on immune-suppressant or steroid drugs; and (6) patients presenting with ST-segment-elevated myocardial infarction (STEMI) (**Figure 1**). A total of 7,826 patients were finally enrolled in this study and were categorized according to the presence of T2D and then by the median value of ANC (4.05 ∗ 10^9^/L), as ANC-L/non-T2D (n = 2,260), ANC-H/non-T2D (n = 1,940), ANC-L/T2D (n = 1,642), and ANC-H/T2D (n = 1,984).

The study process complied with the Declaration of Helsinki and was approved by the Institutional Review Board of Fuwai Hospital. Written informed consent for long-term follow-up before PCI was obtained from all study patients.

### Procedure and medications

PCI procedures were according to standard techniques, and the choice of devices and auxiliary examinations, including intravascular ultrasound and optical coherence tomography, was at the discretion of the interventionalists. Before PCI, unless already on dual antiplatelet therapy, patients were treated with a 300-mg loading dose of aspirin and a 180-mg loading dose of ticagrelor or a 300-mg loading dose of clopidogrel. The periprocedural administration of antiplatelet and antithrombotic medications was according to the guidelines in effect at the time. After the procedure, patients were prescribed aspirin (100 mg daily) indefinitely and ticagrelor (90 mg, twice daily) or clopidogrel (75 mg, daily) for at least 12 months. The data were entered into a dedicated database by independent research personnel (15).

### Clinical assessment and angiographic information

Baseline characteristics based on demographic information, individual health habits, medical history, and drug prescriptions were acquired from each patient by well-trained cardiologists. T2D was defined as FBG ≥7.0 mmol/L (126 mg/dl), HbA1c ≥6.5%, 2-h blood glucose from an oral glucose tolerance test ≥11.1 mmol/L (200 mg/dl), or a previous definite diagnosis of T2D with hypoglycemic drugs or insulin treatment (16). Hypertension was defined as self-reported hypertension with antihypertensive drugs or newly recorded systolic blood pressure ≥140 mmHg and/or diastolic blood pressure ≥90 mmHg for three or more consecutive times on different days. According to previous studies (15), dyslipidemia was diagnosed by fasting total cholesterol (TC) ≥5.2 mmol/L, low-density lipoprotein cholesterol (LDL-C) ≥3.4 mmol/L, high-density lipoprotein cholesterol (HDL-C) <1.0 mmol/L, or triglycerides (TG) ≥1.7 mmol/L, and/or receiving lipid-lowering medications. Kidney dysfunction was considered when the estimated glomerular filtration rate (eGFR) was less than 90 ml/min ∗ 1.73 m^2^ (17). Body mass index (BMI) was calculated by weight (in kg) divided by height (in meters)^2^.

Coronary angiograms were independently evaluated at the angiographic core laboratory of Fuwai Hospital for the process of coronary angiology and stenting procedures and recorded the number of coronary arteries with stenosis of 50% or more, special types of coronary stenotic lesions [such as left main (LM) disease, three-vessel disease, and chronic total occlusion (CTO) disease], the Synergy Between PCI with TAXUS and Cardiac Surgery (SYNTAX) score, and the number of treated vessels and implanted stents.

### Laboratory measurements

After at least a 12-hour fast, blood samples were routinely collected from each patient on the day of admission. A complete blood count, including neutrophils, lymphocytes, and platelets, was performed using an automatic blood cell analyzer (XT-1800i; Sysmex Corporation). The HbA1c levels were estimated by the Tosoh Automated Glycohemoglobin Analyzer (HLC-723G8, Tokyo, Japan). Other laboratory parameters, including lipid profiles (TG, TC, HDL-C, and LDL-C), FBG, creatinine, and high-sensitivity CRP (hsCRP), were examined in the core laboratory at Fuwai Hospital, according to the standard procedures in effect at the time (15). Calculation of eGFR was performed using the Chinese-modified MDRD (Modification of Diet in Renal Disease) equation (18). Based on a modified Simpson’s rule, the left ventricular ejection fraction (LVEF) was estimated from two-dimensional echocardiography.

### Follow-up and study endpoints

The clinical status was collected at 1, 6, and 12 months and yearly thereafter by an independent group of professional clinical research coordinators through telephone contacts or outpatient visits. The primary endpoint was major adverse cardiovascular and cerebrovascular events (MACCEs), defined as a composite of all-cause mortality, myocardial infarction (MI), stroke, and target vessel revascularization (TVR). The secondary end points included individual components of MACCEs. Consistent with our prior research (15), all-cause mortality was defined as mortality from any cause. MI was defined according to the Third Universal Definition of MI (19). Stroke was defined as neurological deficits, either ischemic or hemorrhagic, confirmed by a neurologist based on imaging findings. TVR was defined as a repeat PCI or coronary artery bypass graft (CABG) in the target vessel.

### Statistical analyses

Continuous variables are expressed as the mean ± standard deviation and were compared by Student’s t-test, Mann–Whitney U test, or analysis of variance as appropriate. Categorical variables are presented as numbers with percentages and are tested by the χ^2^ test or Fisher’s exact test. In survival analysis, restricted cubic splines (RCSs) were created to assess the linearity assumptions of the association between ANC and MACCEs. The cumulative incidence of MACCEs among groups was estimated by Kaplan–Meier analysis and compared by the log-rank test. The proportional hazards assumption was assessed by Schoenfeld residuals. Univariable and multivariable Cox proportional hazard analyses were performed to estimate the association of ANC and T2D with the risk of adverse clinical events. Hazard ratios (HRs) and 95% confidence intervals (CIs) were presented. Variables, including age, male sex, BMI, hypertension, dyslipidemia, smoking history, previous MI, previous PCI/CABG, previous stroke, non-ST-segment-elevated acute coronary syndrome (NSTE-ACS), hsCRP, eGFR, LVEF, LM/three-vessel disease, CTO lesions, number of treated vessels, and SYNTAX score, were included in the multivariable Cox model for their statistical significance in univariable analysis or clinical importance in clinical trials. Additionally, compared with the predictive model containing clinical risk factors, the additional predictive performance of the new classification by ANC categories and T2D was determined by the receiver operating characteristic (ROC) analysis with area under the ROC curve (AUC), the category-free net reclassification index (NRI), and the integrated discrimination improvement (IDI). Furthermore, subgroup analyses were adopted to evaluate the risk of MACCEs according to six different subsets and illustrated as forest plots. A two-tailed P-value of <0.05 was considered statistically significant. Statistical analyses were performed using RStudio software (version 2021.09.0; http://www.rstudio.org/).

## Results

### Baseline characteristics

In general, a total of 7,826 CAD patients (58.49 ± 10.15 years, 75.4% male) who received PCI with DES implantation were consecutively enrolled in this study. During a median follow-up of 2.4 years, 69 (0.9%) all-cause mortality, 47 (0.6%) MIs, 111 (1.4%) strokes, 330 (4.2%) TVRs, and 509 (6.5%) MACCEs were observed.

**Table 1** illustrates the baseline characteristics of the study population stratified by the occurrence of MACCEs. Individuals with any component of MACCEs tended to be older with a higher prevalence of T2D, previous MI, and previous stroke compared with those in the non-MACCE group. In addition, higher ANC, FBG, HbA1c, and hsCRP levels and lower LVEF levels were noted in patients who experienced MACCEs. Regarding angiographic information, LM/three-vessel disease and CTO lesions were more prevalent in patients who suffered MACCEs, with a higher SYNTAX score, number of treated vessels, and number of stents.

**Table 1:** Baseline characteristics for individuals with or without MACCEs.

Variables	Overall (N = 7,826)	Non-MACCE (N = 7,317)	MACCE (N = 509)	P-value
Risk groups				<0.001
ANC-L/Non-T2D	2,260 (28.9)	2,139 (29.2)	121 (23.8)	
ANC-H/Non-T2D	1,940 (24.8)	1,839 (25.1)	101 (19.8)	
ANC-L/T2D	1,642 (21.0)	1,538 (21.0)	104 (20.4)	
ANC-H/T2D	1,984 (25.4)	1,801 (24.6)	183 (36.0)	
Baseline characteristics				
Age, years	58.49 ± 10.15	58.39 ± 10.12	59.85 ± 10.50	0.002
Male	5,904 (75.4)	5,512 (75.3)	392 (77.0)	0.424
BMI, kg/m^2^	25.92 ± 3.20	25.93 ± 3.20	25.89 ± 3.18	0.819
T2D	3,626 (46.3)	3,339 (45.6)	287 (56.4)	<0.001
Hypertension	5,100 (65.2)	4,750 (64.9)	350 (68.8)	0.087
Dyslipidemia	5,216 (66.6)	4,858 (66.4)	358 (70.3)	0.076
Smoking history	4,378 (55.9)	4,097 (56.0)	281 (55.2)	0.765
Previous MI	460 (5.9)	413 (5.6)	47 (9.2)	0.001
Previous PCI/CABG	1,652 (21.1)	1,527 (20.9)	125 (24.6)	0.055
Previous stroke	819 (10.5)	745 (10.2)	74 (14.5)	0.002
Previous PAD	585 (7.5)	548 (7.5)	37 (7.3)	0.924
Clinical presentation				0.759
SAP	2,871 (36.7)	2,688 (36.7)	183 (36.0)	
NSTE-ACS	4,955 (63.3)	4629 (63.3)	326 (64.0)	
Laboratory tests				
Neutrophils, ∗10^9^/L	4.37 ± 1.76	4.36 ± 1.75	4.62 ± 1.94	0.001
Lymphocytes, ∗10^9^/L	1.95 ± 0.65	1.95 ± 0.65	1.98 ± 0.67	0.279
Platelet, ∗10^9^/L	174.47 ± 86.78	174.09 ± 86.96	180.02 ± 84.03	0.148
FBG, mmol/L	6.32 ± 2.25	6.30 ± 2.25	6.60 ± 2.21	0.004
HbA1c, %	6.60 ± 1.23	6.59 ± 1.23	6.79 ± 1.28	<0.001
TG, mmol/L	1.78 ± 1.09	1.79 ± 1.09	1.76 ± 1.10	0.288
TC, mmol/L	4.24 ± 1.07	4.24 ± 1.08	4.22 ± 1.03	0.636
HDL-C, mmol/L	1.04 ± 0.28	1.04 ± 0.28	1.03 ± 0.28	0.424
LDL-C, mmol/L	2.53 ± 0.90	2.53 ± 0.91	2.52 ± 0.86	0.680
hsCRP, mg/L	3.13 ± 3.68	3.11 ± 3.67	3.47 ± 3.84	0.019
Creatinine, μmol/L	74.85 ± 15.32	74.77 ± 15.27	76.08 ± 15.84	0.061
eGFR, mL/min/1.73 m2	103.06 ± 22.15	103.18 ± 22.05	101.39 ± 23.43	0.077
LVEF, %	63.75 ± 6.49	63.80 ± 6.46	63.16 ± 6.87	0.034
Medications				
Aspirin	7,730 (98.8)	7,226 (98.8)	504 (99.0)	0.757
Clopidogrel	7,727 (98.7)	7,224 (98.7)	503 (98.8)	1.000
β-blocker	7,020 (89.7)	6,551 (89.5)	469 (92.1)	0.072
CCB	4,010 (51.2)	3,742 (51.1)	268 (52.7)	0.539
Statins	7,505 (95.9)	7,019 (95.9)	486 (95.5)	0.708
Nitrate	7,662 (97.9)	7,165 (97.9)	497 (97.6)	0.790
Coronary procedural data				
LM/three-vessel disease	3,363 (43.0)	3,095 (42.3)	268 (52.7)	<0.001
CTO lesions	543 (6.9)	496 (6.8)	47 (9.2)	0.044
Bifurcation lesions	1,566 (20.0)	1,476 (20.2)	90 (17.7)	0.193
Number of treated vessels	1.41 ± 0.66	1.40 ± 0.66	1.49 ± 0.71	0.003
Number of stents	1.90 ± 1.04	1.89 ± 1.05	1.98 ± 1.02	0.017
SYNTAX score	11.78 ± 7.63	11.70 ± 7.59	12.95 ± 8.12	0.001

Values are mean ± standard deviation or n (%).

MACCE, major adverse cardiovascular and cerebrovascular events; ANC, absolute neutrophil counts; T2D, type 2 diabetes; BMI, body mass index; MI, myocardial infarction; PCI, percutaneous coronary intervention; CABG, coronary artery bypass grafting; PAD, peripheral artery disease; COPD, chronic obstructive pulmonary disease; SAP, stable angina pectoris; NSTE-ACS, non-ST-segment elevation acute coronary syndrome; FBG, fasting blood glucose; HbA1c, glycosylated hemoglobin A1c; TG, triglyceride; TC, total cholesterol; HDL-C, high-density lipoprotein cholesterol; LDL-C, low-density lipoprotein cholesterol; hsCRP, high-sensitivity C-reactive protein; eGFR, estimated glomerular filtration rate; LVEF, left ventricular ejection fraction; CCB, calcium channel blocker; LM, left main; CTO, chronic total occlusion; SYNTAX, synergy between PCI with taxus and cardiac surgery.

### Baseline characteristics of individuals in four risk groups

According to the ANC median value of 4.05 ∗ 10^9^/L and T2D, patients were stratified into four risk groups. Subsequently, the baseline characteristics of the four risk groups are presented in **Table 2**.

**Table 2:** Baseline characteristics for diabetic or non-diabetic patients with different ANC levels.

Variables	ANC-L/Non-T2D (n = 2,260)	ANC-H/Non-T2D (n = 1,940)	ANC-L/T2D (n = 1,642)	ANC-H/T2D (n = 1,984)	P-value
Baseline characteristics					
Age, years	58.30 ± 10.08	56.83 ± 10.40	59.94 ± 9.37	59.12 ± 10.36	<0.001
Male	1,664 (73.6)	1,601 (82.5)	1,137 (69.2)	1,502 (75.7)	<0.001
BMI, kg/m^2^	25.41 ± 3.13	25.88 ± 3.26	26.04 ± 3.04	26.46 ± 3.25	<0.001
DM (%)	0 (0.0)	0 (0.0)	1,642 (100.0)	1,984 (100.0)	<0.001
Hypertension	1,339 (59.2)	1,247 (64.3)	1,113 (67.8)	1,401 (70.6)	<0.001
Dyslipidemia	1,402 (62.0)	1,251 (64.5)	1,176 (71.6)	1,387 (69.9)	<0.001
Smoking history	1,151 (50.9)	1,236 (63.7)	812 (49.5)	1,179 (59.4)	<0.001
Previous MI	128 (5.7)	86 (4.4)	110 (6.7)	136 (6.9)	0.005
Previous PCI/CABG	421 (18.6)	317 (16.3)	395 (24.1)	519 (26.2)	<0.001
Previous stroke	178 (7.9)	176 (9.1)	213 (13.0)	252 (12.7)	<0.001
Previous PAD	146 (6.5)	123 (6.3)	158 (9.6)	158 (8.0)	<0.001
Clinical presentation					<0.001
SAP	890 (39.4)	603 (31.1)	704 (42.9)	674 (34.0)	
NSTE-ACS	1,370 (60.6)	1,337 (68.9)	938 (57.1)	1,310 (66.0)	
Laboratory tests					
Neutrophils, ∗10^9^/L	3.13 ± 0.60	5.40 ± 1.43	3.20 ± 0.57	5.77 ± 1.92	<0.001
Lymphocytes, ∗10^9^/L	1.82 ± 0.55	2.00 ± 0.66	1.91 ± 0.61	2.07 ± 0.73	<0.001
Platelet, ∗10^9^/L	164.73 ± 79.76	185.45 ± 91.30	161.19 ± 81.02	185.83 ± 91.57	<0.001
FBG, mmol/L	5.12 ± 0.55	5.23 ± 0.61	7.24 ± 2.36	8.00 ± 2.89	<0.001
HbA1c, %	5.86 ± 0.34	5.88 ± 0.33	7.41 ± 1.32	7.47 ± 1.37	<0.001
TG, mmol/L	1.59 ± 0.87	1.81 ± 1.00	1.85 ± 1.31	1.93 ± 1.17	<0.001
TC, mmol/L	4.19 ± 1.03	4.26 ± 1.05	4.21 ± 1.13	4.30 ± 1.10	0.003
HDL-C, mmol/L	1.09 ± 0.29	1.03 ± 0.28	1.04 ± 0.29	1.00 ± 0.25	<0.001
LDL-C, mmol/L	2.50 ± 0.88	2.55 ± 0.90	2.49 ± 0.93	2.59 ± 0.92	0.002
hsCRP, mg/L	2.08 ± 2.66	3.83 ± 4.11	2.42 ± 2.95	4.34 ± 4.33	<0.001
Creatinine, μmol/L	73.21 ± 13.64	76.35 ± 14.85	72.75 ± 15.15	77.00 ± 17.16	<0.001
eGFR, mL/min/1.73 m^2^	104.70 ± 20.57	102.42 ± 21.09	104.88 ± 23.23	100.33 ± 23.62	<0.001
LVEF, %	64.56 ± 5.91	63.75 ± 6.54	64.17 ± 6.35	62.50 ± 6.99	<0.001
Medications					
Aspirin	2,232 (98.8)	1,912 (98.6)	1,628 (99.1)	1,958 (98.7)	0.429
Clopidogrel	2,225 (98.5)	1,915 (98.7)	1,624 (98.9)	1,963 (98.9)	0.472
β-blocker	1,983 (87.7)	1,719 (88.6)	1,492 (90.9)	1,826 (92.0)	<0.001
CCB	1,126 (49.8)	970 (50.0)	861 (52.4)	1,053 (53.1)	0.085
Statins	2,191 (96.9)	1,864 (96.1)	1,556 (94.8)	1,894 (95.5)	0.005
Nitrate	2,209 (97.7)	1,917 (98.8)	1,604 (97.7)	1,932 (97.4)	0.011
Procedural data					
LM/three-vessel disease	858 (38.0)	755 (38.9)	771 (47.0)	979 (49.3)	<0.001
CTO lesions	163 (7.2)	129 (6.6)	114 (6.9)	137 (6.9)	0.915
Bifurcation lesions	457 (20.2)	397 (20.5)	312 (19.0)	400 (20.2)	0.709
Number of treated vessels	1.37 ± 0.62	1.40 ± 0.65	1.41 ± 0.65	1.47 ± 0.72	<0.001
Number of stents	1.83 ± 1.01	1.88 ± 1.01	1.92 ± 1.07	1.97 ± 1.08	<0.001
SYNTAX score	10.89 ± 7.20	11.72 ± 7.59	11.93 ± 7.61	12.73 ± 8.03	<0.001

Values are mean ± standard deviation or n (%).

Abbreviations as in **Table 1**.

Diabetic patients with a higher level of ANC were older and more likely to be female, with a greater burden of concomitant diseases, including hypertension, dyslipidemia, smoking history, previous MI, previous stroke, and previous peripheral artery disease (PAD), and clinical presentations as NSTE-ACS compared with those in other groups. Additionally, a higher prevalence of prior PCI/CABG and LM/three-vessel disease was observed in the ANC-H/T2D group. Moreover, there was also higher BMI, FBG, HbA1c, TG, TC, LDL-C, hsCRP, creatinine, number of treated vessels, number of stents, and SYNTAX score, and lower HDL-C, eGFR, and LVEF in the ANC-H/T2D group.

### Association of ANC and type 2 diabetes with clinical outcomes

During a median follow-up of 2.4 years, the incidence of MACCE in the ANC-L/Non-T2D, ANC-H/Non-T2D, ANC-L/T2D, and ANC-H/T2D groups was 5.4% (121/2260), 5.2% (101/1940), 6.3% (104/1642), and 9.2% (183/1984), respectively.

**Figure 2:**
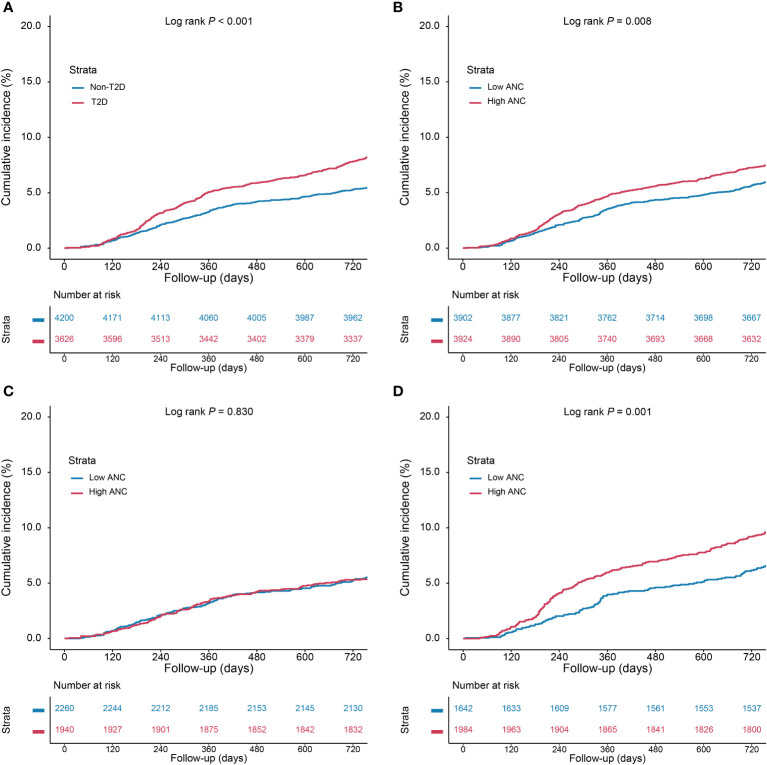
Kaplan–Meier (KM) curves for cumulative incidence of MACCEs. **(A)** KM curves according to type 2 diabetes, **(B)** KM curves according to ANC categories, **(C)** KM curves according to ANC categories in non-diabetic patients, and **(D)** KM curves according to ANC categories in diabetic patients. Abbreviations as in **Figure 1**.

As depicted in **Figure 2**, the Kaplan–Meier curves revealed that T2D and a higher level of ANC conferred a higher risk of MACCEs compared with the other groups (Log rank P <0.001 and P = 0.008, respectively). In addition, there was a greater rate of MACCEs in patients with higher levels of ANC among diabetic patients (log rank P = 0.001), whereas no significant difference in MACCE risk was observed among non-diabetic patients (log rank P = 0.830). **Table 3** displays the results of the Cox proportional analysis evaluating the association of ANC and glycemic status with clinical outcomes. A significant interaction was observed between T2D and ANC categories after adjusting for age, male sex, BMI, hypertension, dyslipidemia, smoking history, previous MI, previous PCI/CABG, previous stroke, NSTE-ACS, hsCRP, eGFR, LVEF, LM/three-vessel disease, CTO lesions, number of treated vessels, and SYNTAX score (P = 0.044 for interaction). In patients without T2D, no significant association between ANC and MACCE risk was detected when evaluated either by ANC [adjusted HR (aHR), 0.99; 95% CI, 0.90–1.09] or a categorical threshold of 4.05 ∗ 10^9^/L (aHR, 0.90; 95% CI, 0.68–1.19). On the other hand, in diabetic patients, elevation of ANC, either by a 1 ∗ 10^9^/L increase (aHR, 1.09; 95% CI, 1.02–1.16; P = 0.017) or a categorical threshold of 4.05 ∗ 10^9^/L (aHR, 1.45; 95% CI, 1.12–1.87; P = 0.005), was significantly associated with a higher risk of MACCEs.

**Table 3:** Prognostic value of ANC for MACCEs according to different glycemic status.

	Events/total	Crude HR (95% CI)	P-value	Adjusted HR (95% CI)*	P-value†
Glycemic status categories					
Non-T2D	222/4,200	Reference	NA	Reference	NA
T2D	287/3,626	1.52 (1.27–1.81)	<0.001	1.37 (1.14–1.65)	0.001
ANC categories					
ANC, per 1 ∗ 10^9^/L	509/7,826	1.08 (1.03–1.12)	<0.001	1.06 (1.00–1.12)	0.045
ANC <4.05 ∗ 10^9^/L	225/3,902	Reference	NA	Reference	NA
ANC ≥4.05 ∗ 10^9^/L	284/3,924	1.27 (1.06–1.51)	0.008	1.19 (0.99–1.44)	0.069
Non-T2D categories					
ANC, per 1 ∗ 10^9^/L	222/4,200	1.01 (0.93–1.10)	0.827	0.99 (0.90–1.09)	0.834
ANC<4.05 ∗ 10^9^/L	121/2,260	Reference	NA	Reference	NA
ANC≥4.05 ∗ 10^9^/L	101/1,940	0.97 (0.75–1.27)	0.830	0.90 (0.68–1.19)	0.457
T2D categories					
ANC, per 1 ∗ 10^9^/L	287/3,626	1.08 (1.03–1.14)	0.002	1.09 (1.02–1.16)	0.017
ANC<4.05 ∗ 10^9^/L	104/1,642	Reference	NA	Reference	NA
ANC≥4.05 ∗ 10^9^/L	183/1,984	1.49 (1.17–1.89)	0.001	1.45 (1.12–1.87)	0.005

*Adjusted for age, male sex, BMI, hypertension, dyslipidemia, smoking history, previous MI, previous PCI/CABG, previous stroke, NSTE-ACS, hsCRP, eGFR, LVEF, LM/three-vessel disease, CTO lesions, number of treated vessels, and SYNTAX score.

†P for interaction for the risk of MACCE: ANC and T2D = 0.380; ANC categories and T2D = 0.044.

HR, harzard ratio; CI, confidence interval; NA, not applicable; other abbreviations as in **Table 1**.

### Risk of adverse events in different risk groups classified by type 2 diabetes and ANC categories

**Figure 3:**
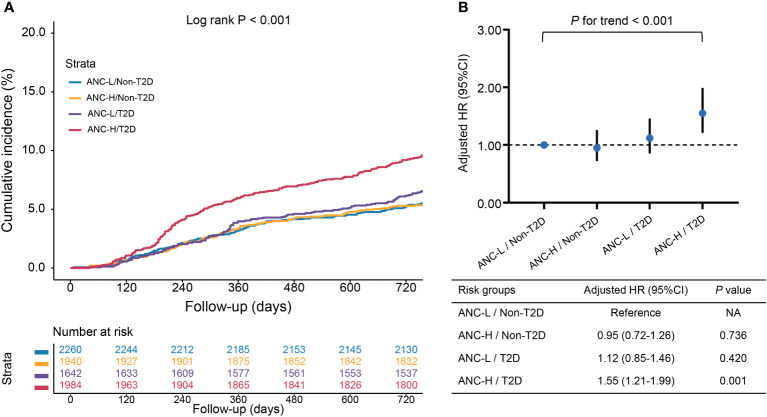
The associations of risk groups with the risk of MACCEs. **(A)** Kaplan–Meier curves for cumulative incidence of MACCEs according to four risk groups; **(B)** Hazard ratios (HRs) [95% confidence intervals (CIs)] for MACCEs according to four risk groups. Model adjusted for age, male sex, BMI, hypertension, dyslipidemia, smoking history, previous MI, previous PCI/CABG, previous stroke, NSTE-ACS, hsCRP, eGFR, LVEF, LM/three-vessel disease, CTO lesions, number of treated vessels, and SYNTAX score. Abbreviations as in **Figure 1**.

As shown in **Figure 3**, the cumulative incidence of the MACCE rate was highest in the ANC-H/T2D group. HRs were estimated for each group using the ANC-L/non-T2D group as a reference (**Additional File 1**: **Tables S1**, **S2**). In fully adjusted analyses, patients in the ANC-H/T2D group were at significantly higher risk of MACCEs (aHR, 1.55; 95% CI, 1.21–1.99; P = 0.001) and TVR (aHR, 1.55; 95% CI, 1.15–2.10; P = 0.004). In addition, multivariable Cox proportional analysis also revealed the highest risk of MACCEs in diabetic patients with a higher level of ANC than others (P <0.001 for trend). RCS analysis exhibited a linear association between ANC and the MACCE risk regardless of the univariable and multivariable model (**Additional file 1**: **Figure S1**; P >0.05 for a non-linear association for both).

In the original model, the AUC was 0.587 (95% CI, 0.562–0.612) with clinical risk factors, including age, male sex, hypertension, dyslipidemia, smoking history, previous stroke, previous MI, NSTE-ACS, LVEF, eGFR, and the SYNTAX score. When we added the combination of ANC categories and T2D to the original model, there was a significant but slight improvement in AUC to 0.607 (95% CI, 0.582–0.632) (ΔAUC, 0.020; P = 0.027) (**Additional File 1**: **Figure S2**).

### Subgroup analysis

**Figure 4:**
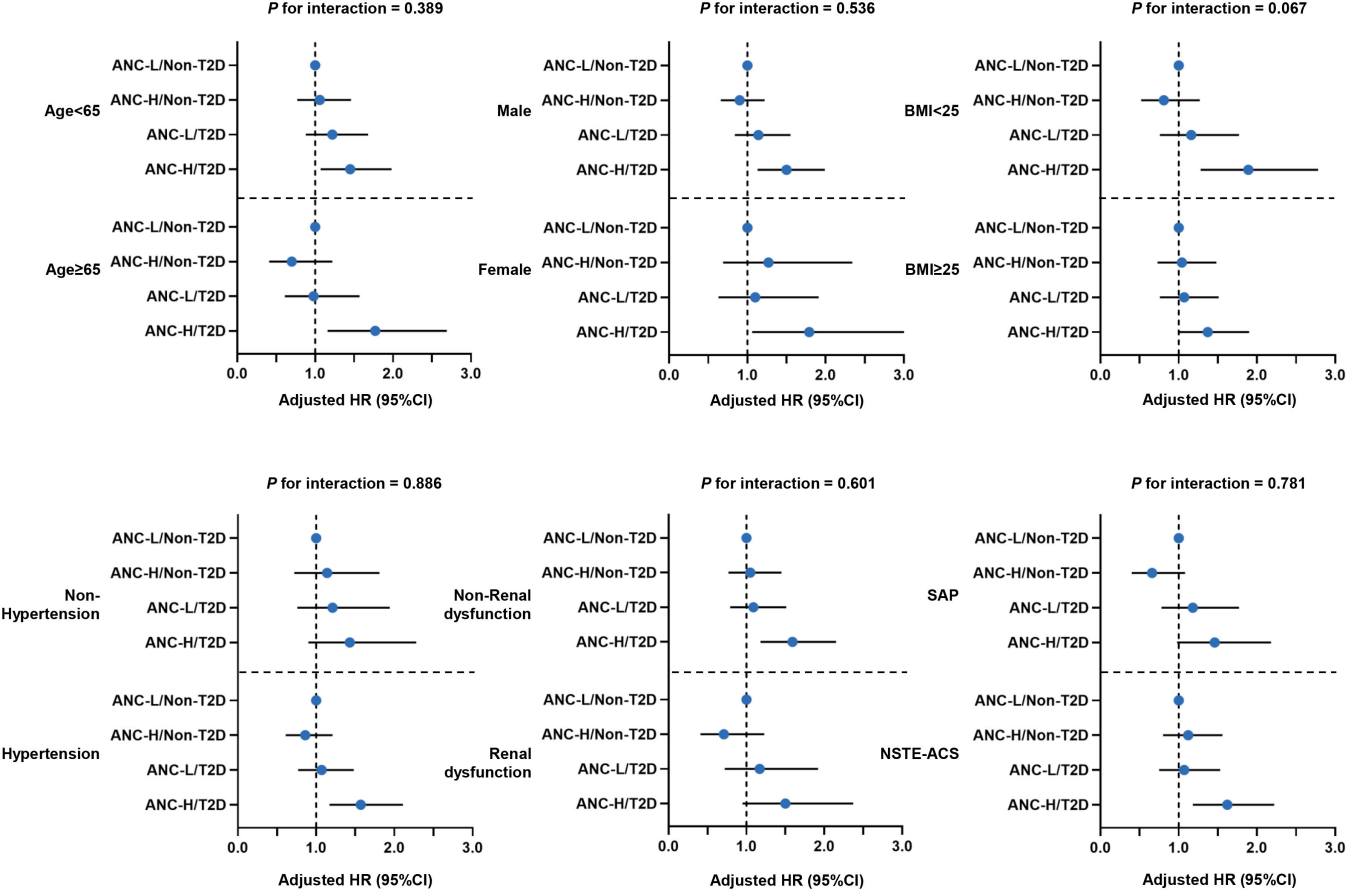
Forest plot of MACCE risk according to various subgroups. Model adjusted for age, male sex, BMI, hypertension, dyslipidemia, smoking history, previous MI, previous PCI/CABG, previous stroke, NSTE-ACS, hsCRP, eGFR, LVEF, LM/three-vessel disease, CTO lesions, number of treated vessels, and SYNTAX score. Abbreviations as in **Figure 1**.

In subgroup analyses, the relationship between four risk groups and MACCE risk was consistent across different subgroups (age, sex, BMI, hypertension, kidney dysfunction, and clinical presentation), with comparable interactions (all P >0.05 for interactions) (**Figure 4**, **Additional File 1**: **Table S3**).

## Discussion

In this real-world, large-scale, prospective, observational cohort study, the prognosis of diabetic and non-diabetic CAD patients undergoing PCI with different levels of ANC was investigated. The major findings of this study are as follows (1): T2D modified the effect of ANC on clinical outcomes in CAD patients who underwent PCI after adjustment for confounding factors. A higher level of ANC was independently associated with MACCEs in T2D. While in non-diabetic patients, no significant association between ANC and MACCE risk was found; (2) elevated ANC with concomitant T2D conferred an increased MACCE risk in CAD patients undergoing PCI; (3) diabetic patients with elevated ANC tended to have unfavorable cardiovascular risk profiles and increased lesion complexity, assessed by the SYNTAX score; and (4) the association of ANC categories and T2D with adverse clinical outcomes was consistent according to different subgroups. Our findings suggested that more precise and accurate risk assessment and appropriate clinical management should be performed in CAD patients with concomitant T2D and elevated ANC.

Current evidence has indicated that inflammation might be an essential and modifiable process in the development of CAD (20). Previous studies reported a range of inflammatory biomarkers that were associated with an increased risk of cardiovascular events (21–23). Among them, white blood cells (WBCs) were one of the simplest and most measured blood-based inflammatory biomarkers. Since Friedman et al. (24) first demonstrated the WBC count could be a predictor of myocardial infarction in 1974, it has been suggested to be positively associated with higher rates of incident CV events and mortality (25, 26). Subsequently, several researchers evaluated the ability of different subpopulations of WBC to estimate the risk of CV events and mortality, which yielded controversial conclusions (26, 27). Wheeler et al. (27) conducted a meta-analysis to investigate the relationship between differential leucocyte count and incidence of CAD in 28,528 patients with or without a history of CAD from five large cohorts with a mean follow-up ranging from 3 to 18 years. Comparing the top tertiles vs. bottom tertiles of different leucocyte counts, they found the association of CAD with ANC was stronger than that of granulocytes, lymphocytes, and monocytes, with a risk ratio of 1.33, 1.32, 1.11, and 1.10, respectively. Besides, Welsh et al. (26) also found a similar conclusion from 478,259 UK biobank participants with a 7-year follow-up. After adjusting for a range of classical risk factors, they found only ANC was most consistently associated with the risk of fatal and nonfatal CVD in both men and women. Furthermore, Shah et al. (28) found strong associations between ANC and heart failure, unheralded coronary death, abdominal aortic aneurysm, nonfatal MI, and ischemic stroke. Overall, these studies indicated ANC had a strong positive association with the risk of all-cause or CV mortality in the general population.

Currently, several studies have explored the impact of neutrophils on CVD risk from biological mechanisms and clinically modifiable risk factors. From a biological standpoint, during the past decades, studies have demonstrated the important role of neutrophils in the process of atherosclerosis and CV inflammation by several mechanisms, as follows (1): In coronary lesions, neutrophils could be observed near plaques, especially rupture-prone ones (29). (2) Neutrophils could enhance the adhesion of monocytes, which might transform into foam cells and form the lipid core of vulnerable plaques (30). Meanwhile, neutrophils also contribute to endothelial cell dysfunction and oxidative stress *via* releasing myeloperoxidase, lipoxygenases, and NETs (30, 31). NETs can also promote atherothrombosis and present tissue factors at sites of infarction (31). (3) Neutrophils could promote fibrous cap rupture by releasing proteases that degrade matrix elements (29). (4) An early massive neutrophil influx into the ischemia-injured myocardium can cause collateral damage and impair myocardial healing (30). On the other hand, from clinical sights, acute inflammation, chronic inflammation background and genetics of patients might help to explain the relationship between ANC and CVD. Of note, low-grade and chronic inflammation might be the initial but modifiable factor in the causal pathways of CVD, including air pollution, obesity, a lack of physical exercise, and periodontal disease (32, 33). Here, we focus on T2D. Previous studies indicated that low-grade inflammation is a key component in the pathophysiology of T2D (34). Meanwhile, increased diabetic risk (35) and insulin resistance (36) have been described in patients with chronic inflammatory diseases such as rheumatoid arthritis and psoriasis. One previous cohort study assessed the role of neutrophils in the pathogenesis of T2D (37). However, no evidence indicated a relationship between neutrophils, T2D, and the risk of CVD. In our study, we found that for T2D patients, a higher level of ANC was independently associated with 2-year MACCEs. However, for patients without T2D, no statistically significant association was observed between ANC and 2-year MACCEs.

The interaction between inflammation, T2D, and CVD might be explained by the co-signaling of IL-1β and IL-6 in diabetes and atherosclerosis. Firstly, IL-1β was one of the key components of inflammatory activation by producing the inflammasome, which could regulate the activation of caspase-1 and mediate NLRP3. For T2D, one previous study had indicated IL-1β could impair insulin secretion, contributing to the development of glucose intolerance and T2D (38). Meanwhile, IL-1β could also promote insulin resistance *via the* NLRP3 inflammasome (39) and impair adipocyte insulin signaling *via the* activation of caspase-1 (40). For atherosclerosis, the formation of foam cells was initiated by the release of IL-1β and NLRP3 inflammasomes, which were activated by cholesterol and saturated fatty acids (41). Meanwhile, IL-β could regulate the chemotaxis and adhesion of monocytes (42). Secondly, current evidence indicates that IL-6 is a central stimulus for the acute phase response, for example by increasing the production of CRP (43). For atherosclerosis, the results from an animal model shows that IL-6 signaling contributed to the initiation of atherosclerosis and plaque destabilization in mice (44). For T2D, current studies have indicated that IL-6 has a negative effect on metabolism. It was supported by elevated circulating IL-6 in patients with obesity and the onset of diabetes (45). Meanwhile, one previous study indicated that IL-6 could induce hepatic insulin resistance (46).

It was the first and largest cohort study to investigate the interaction between neutrophils, T2D, and poor prognosis in CAD patients. However, there were still some limitations. First, even though the present study adjusted for a considerable number of potential confounding factors, it was hard to fully adjust all confounding factors (such as diet, air pollution, and physical exercise) due to the limitations of data collection and the nature of the observational study design. Second, data regarding dynamic changes in ANC and glycemic status during follow-up are unavailable. Thirdly, the ethnic composition of this study was single, only Chinese patients were included (47, 48). It was still unclear whether the conclusion was suitable for other populations. Fourth, in this study, we performed multivariable Cox analysis to adjust for potential confounding factors. However, a sensitivity analysis (propensity score matching or a nested case–control design) was not adopted to strengthen our results. Future, well-designed prospective studies are warranted to confirm our findings.

## Conclusions

Elevated ANC confers increased MACCE risk with T2D. Patients with concomitantly elevated ANC and T2D have greater MACCE risk; stratification of patients with elevated ANC and T2D could provide valuable information for risk stratification of CAD patients.

## Data availability statement

The original contributions presented in the study are included in the article/**Supplementary Material**. Further inquiries can be directed to the corresponding authors.

## Ethics statement

The study process complied with the Declaration of Helsinki and was approved by the Institutional Review Board of Fuwai hospital. Written informed consent for long term follow-up before PCI was obtained from all study patients.

## Author contributions

KD and QD conceived and designed the study. JH, ZL, CS, and RZ acquired, analyzed, and interpreted the data. JH, ZL, and SY contributed to drafting the article. JH, ZL, HW, and XB revised it critically for important intellectual content. All authors listed have made a substantial, direct, and intellectual contribution to the work and approved it for publication.
