# Supplementary material for: High absolute neutrophil count with type 2 diabetes is associated with adverse outcome in patients with coronary artery disease: A large-scale cohort study
Source: Front Endocrinol (Lausanne). 2023 Apr 11;14:1129633. doi: 10.3389/fendo.2023.1129633 (PMC10126907; doi:10.3389/fendo.2023.1129633)
Supplement: Supplementary file 1 [file DataSheet_1.docx]

**High Absolute Neutrophil Count with Type 2 Diabetes is Associated with Adverse Outcome in Patients Undergoing Percutaneous Coronary Intervention: A Large-Scale Cohort Study**

**Supplementary materials**

| **Table of Contents** | | **Page Number** |
| --- | --- | --- |
| Table S1 | Univariable and Multivariable Cox proportional hazard analysis for primary endpoint | 2-3 |
| Table S2 | Predictive value of the ANC levels and different glycemic metabolism status for MACCEs in univariable and multivariable analysis | 4 |
| Table S3 | Subgroup analyses for the primary endpoint as the adjusted model | 5 |
| Fig. S1 | Restricted cubic splines of ANC levels in relation to crude HR (A) and adjusted HR(B) for the risk of MACCE | 6 |
| Fig. S2 | Comparison of discrimination and reclassification ability of predictive models for MACCEs during follow-up | 7 |

**Table S1** Univariable Multivariable Cox proportional hazard analysis for primary endpoint

| **Variable** | **Crude HR** | **95%CI** | **P value** | **Adjusted HR** | **95%CI** | **P value** |
| --- | --- | --- | --- | --- | --- | --- |
| Risk groups |  |  |  |  |  |  |
| ANC-L/Non-T2D | Reference | NA | NA | Reference | NA | NA |
| ANC-H/Non-T2D | 0.97 | 0.75-1.27 | 0.830 | 0.95 | 0.72-1.26 | 0.736 |
| ANC-L/T2D | 1.18 | 0.91-1.54 | 0.206 | 1.12 | 0.85-1.46 | 0.420 |
| ANC-H/T2D | 1.76 | 1.40-2.21 | <0.001 | 1.55 | 1.21-1.99 | 0.001 |
| Age, per 1 year | 1.01 | 1.01-1.02 | 0.002 | 1.01 | 1.00-1.02 | 0.068 |
| Male sex | 1.09 | 0.89-1.34 | 0.410 | 1.23 | 0.96-1.59 | 0.103 |
| BMI, per 1 kg/m2 | 1.00 | 0.97-1.02 | 0.812 | 0.99 | 0.96-1.02 | 0.544 |
| Hypertension | 1.18 | 0.98-1.42 | 0.082 | 1.08 | 0.88-1.33 | 0.445 |
| Dyslipidemia | 1.19 | 0.98-1.44 | 0.074 | 1.15 | 0.94-1.41 | 0.168 |
| Smoking history | 0.97 | 0.82-1.16 | 0.742 | 0.86 | 0.70-1.06 | 0.166 |
| Previous MI | 1.66 | 1.23-2.24 | <0.001 | 1.37 | 0.98-1.91 | 0.068 |
| Previous PCI/CABG | 1.23 | 1.00-1.50 | 0.047 | 1.09 | 0.87-1.37 | 0.474 |
| Previous stroke | 1.48 | 1.15-1.89 | 0.002 | 1.28 | 0.98-1.66 | 0.069 |
| Previous PAD | 0.97 | 0.69-1.35 | 0.851 |  |  |  |
| Clinical presentation as NSTE-ACS | 1.03 | 0.86-1.24 | 0.732 | 1.07 | 0.89-1.30 | 0.473 |
| Neutrophils, per 1*10^9^/L | 1.07 | 1.03-1.12 | <0.001 |  |  |  |
| Lymphocytes, per 1*10^9^/L | 1.07 | 0.94-1.22 | 0.281 |  |  |  |
| Platelet, per 1*10^9^/L | 1.00 | 1.00-1.00 | 0.140 |  |  |  |
| FBG, per 1 mmol/L | 1.05 | 1.02-1.09 | 0.003 |  |  |  |
| HbA1c, per 1 % | 1.12 | 1.05-1.19 | <0.001 |  |  |  |
| TG, per 1 mmol/L | 0.98 | 0.90-1.07 | 0.665 |  |  |  |
| TC, per 1 mmol/L | 0.98 | 0.90-1.06 | 0.640 |  |  |  |
| HDL-C, per 1 mmol/L | 0.88 | 0.64-1.21 | 0.431 |  |  |  |
| LDL-C, per 1 mmol/L | 0.98 | 0.89-1.08 | 0.674 |  |  |  |
| hsCRP, per 1 mg/L | 1.02 | 1.00-1.05 | 0.037 | 1.01 | 0.99-1.04 | 0.291 |
| Creatinine, per 1 μmol/L | 1.01 | 1.00-1.01 | 0.062 |  |  |  |
| eGFR, per 1 mL/min/1.73 m^2^ | 1.00 | 0.99-1.00 | 0.075 | 1.00 | 1.00-1.00 | 0.880 |
| LVEF, per 1 % | 0.99 | 0.97-1.00 | 0.031 | 1.00 | 0.98-1.01 | 0.703 |
| Aspirin | 1.25 | 0.52-3.02 | 0.617 |  |  |  |
| Clopidogrel | 1.08 | 0.48-2.41 | 0.859 |  |  |  |
| β-blocker | 1.36 | 0.99-1.88 | 0.061 |  |  |  |
| CCB | 1.06 | 0.89-1.26 | 0.529 |  |  |  |
| Statins | 0.89 | 0.59-1.36 | 0.601 |  |  |  |
| Nitrate | 0.89 | 0.50-1.57 | 0.682 |  |  |  |
| LM/three-vessel disease | 1.50 | 1.26-1.79 | <0.001 | 1.29 | 1.05-1.59 | 0.014 |
| CTO lesions | 1.39 | 1.03-1.88 | 0.030 | 1.26 | 0.91-1.74 | 0.154 |
| Bifurcation lesions | 0.86 | 0.68-1.07 | 0.179 |  |  |  |
| Number of treated vessels, per 1 vessel | 1.19 | 1.06-1.34 | 0.004 | 1.06 | 0.93-1.22 | 0.364 |
| Number of stents, per 1 stent | 1.07 | 0.99-1.16 | 0.070 |  |  |  |
| SYNTAX score, per 1 point | 1.02 | 1.01-1.03 | <0.001 | 1.01 | 0.99-1.02 | 0.417 |

HR, hazard ratio; CI, confidence interval; ANC, absolute neutrophil counts; T2D, type 2 diabetes; BMI, body mass index; MI, myocardial infarction; PCI, percutaneous coronary intervention; CABG, coronary artery bypass grafting; PAD, peripheral artery disease; COPD, chronic obstructive pulmonary disease; SAP, stable angina pectoris; NSTE-ACS, non-ST-segment elevation acute coronary syndrome; FBG, fasting blood glucose; HbA1c, glycosylated hemoglobin A1c; TG, triglyceride; TC, total cholesterol; HDL-C, high-density lipoprotein cholesterol; LDL-C, low-density lipoprotein cholesterol; hsCRP, high-sensitivity C-reactive protein; eGFR, estimated glomerular filtration rate; LVEF, left ventricular ejection fraction; CCB, calcium channel blocker; LM, left main; CTO, chronic total occlusion; SYNTAX, synergy between PCI with taxus and cardiac surgery.

**Table S2** Predictive value of the ANC levels and different glycemic metabolism status for MACCEs in univariable and multivariable analysis

| **Groups** | **Events/subjects** | **Univariable** | | **Multivariable** | |
| --- | --- | --- | --- | --- | --- |
|  |  | **HR (95%CI)** | ***P* value** | **HR (95%CI)*** | ***P* value** |
| MACCE |  |  |  |  |  |
| ANC-L/Non-T2D | 121/2260 | Reference | NA | Reference | NA |
| ANC-H/Non-T2D | 101/1940 | 0.97 (0.75-1.27) | 0.830 | 0.95 (0.72-1.26) | 0.736 |
| ANC-L/T2D | 104/1642 | 1.18 (0.91-1.54) | 0.206 | 1.12 (0.85-1.46) | 0.420 |
| ANC-H/T2D | 183/1984 | 1.76 (1.40- 2.21) | <0.001 | 1.55 (1.21-1.99) | 0.001 |
| All-cause mortality |  |  |  |  |  |
| ANC-L/Non-T2D | 15/2260 | Reference | NA | Reference | NA |
| ANC-H/Non-T2D | 17/1940 | 1.32 (0.65-2.65) | 0.432 | 1.29 (0.63-2.65) | 0.485 |
| ANC-L/T2D | 11/1642 | 1.01 (0.46-2.19) | 0.985 | 0.96 (0.44-2.10) | 0.909 |
| ANC-H/T2D | 26/1984 | 1.98 (1.05-3.74) | 0.035 | 1.90 (0.97-3.71) | 0.061 |
| Myocardial infarction |  |  |  |  |  |
| ANC-L/Non-T2D | 8/2260 | Reference | NA | Reference | NA |
| ANC-H/Non-T2D | 11/1940 | 1.60 (0.65-3.99) | 0.309 | 1.54 (0.60-3.96) | 0.374 |
| ANC-L/T2D | 12/1642 | 2.07 (0.84-5.05) | 0.112 | 1.73 (0.70-4.27) | 0.238 |
| ANC-H/T2D | 16/1984 | 2.29 (0.98-5.35) | 0.056 | 1.73 (0.70-4.26) | 0.236 |
| Stroke |  |  |  |  |  |
| ANC-L/Non-T2D | 25/2260 | Reference | NA | Reference | NA |
| ANC-H/Non-T2D | 22/1940 | 1.03 (0.58-1.82) | 0.930 | 1.04 (0.57-1.89) | 0.901 |
| ANC-L/T2D | 26/1642 | 1.43 (0.83-2.48) | 0.202 | 1.32 (0.75-2.31) | 0.338 |
| ANC-H/T2D | 38/1984 | 1.74 (1.05-2.88) | 0.032 | 1.33 (0.76-2.35) | 0.319 |
| TVR |  |  |  |  |  |
| ANC-L/Non-T2D | 83/2260 | Reference | NA | Reference | NA |
| ANC-H/Non-T2D | 63/1940 | 0.88 (0.64-1.23) | 0.458 | 0.87 (0.62-1.23) | 0.433 |
| ANC-L/T2D | 65/1642 | 1.08 (0.78-1.49) | 0.653 | 1.04 (0.74-1.45) | 0.827 |
| ANC-H/T2D | 119/1984 | 1.66 (1.25-2.20) | <0.001 | 1.55 (1.15-2.10) | 0.004 |

*Model adjusted for age, male sex, BMI, hypertension, dyslipidemia, smoking history, previous MI, previous PCI/CABG, previous stroke, NSTE-ACS, hsCRP, eGFR, LVEF, LM/three-vessel disease, CTO lesions, number of treated vessels, and SYNTAX score.

Abbreviations as in Table S1.

**Table S3** Subgroup analyses for the primary endpoint as the adjusted model

| **Variables** | **ANC-L/Non-T2D** | **ANC-H/Non-T2D** | **ANC-L/T2D** | **ANC-H/T2D** | **P for interaction** |
| --- | --- | --- | --- | --- | --- |
| **Age** |  |  |  |  | 0.389 |
| <65 | 1.00 (reference) | 1.06 (0.77-1.46) | 1.22 (0.88-1.68) | 1.45 (1.07-1.98) |  |
| ≥65 | 1.00 (reference) | 0.70 (0.41-1.22) | 0.98 (0.61-1.57) | 1.77 (1.16-2.69) |  |
| **Sex** |  |  |  |  | 0.536 |
| Male | 1.00 (reference) | 0.90 (0.66-1.22) | 1.14 (0.84-1.55) | 1.50 (1.13-1.99) |  |
| Female | 1.00 (reference) | 1.27 (0.69-2.34) | 1.10 (0.63-1.91) | 1.79 (1.06-3.03) |  |
| **BMI** |  |  |  |  | 0.067 |
| <25 | 1.00 (reference) | 0.81 (0.52-1.27) | 1.16 (0.76-1.77) | 1.89 (1.28-2.78) |  |
| ≥25 | 1.00 (reference) | 1.04 (0.73-1.48) | 1.07 (0.76-1.51) | 1.37 (0.99-1.90) |  |
| **Hypertension** |  |  |  |  | 0.886 |
| No | 1.00 (reference) | 1.14 (0.72-1.81) | 1.21 (0.76-1.94) | 1.43 (0.90-2.28) |  |
| Yes | 1.00 (reference) | 0.86 (0.61-1.21) | 1.07 (0.77-1.48) | 1.57 (1.17-2.11) |  |
| **Renal dysfunction** |  |  |  |  | 0.601 |
| No | 1.00 (reference) | 1.05 (0.77-1.45) | 1.09 (0.79-1.51) | 1.59 (1.18-2.15) |  |
| Yes | 1.00 (reference) | 0.71 (0.41-1.23) | 1.17 (0.72-1.92) | 1.50 (0.95-2.37) |  |
| **Clinical presentation** |  |  |  |  | 0.781 |
| SAP | 1.00 (reference) | 0.66 (0.40-1.08) | 1.18 (0.78-1.77) | 1.46 (0.98-2.18) |  |
| NSTE-ACS | 1.00 (reference) | 1.12 (0.80-1.56) | 1.07 (0.75-1.53) | 1.62 (1.18-2.22) |  |

Values are presented with HR (95% CI).

Model adjusted for age, male sex, BMI, hypertension, dyslipidemia, smoking history, previous MI, previous PCI/CABG, previous stroke, NSTE-ACS, hsCRP, eGFR, LVEF, LM/three-vessel disease, CTO lesions, number of treated vessels, and SYNTAX score.

Abbreviations as in Table S1.

**Fig. S1** Restricted cubic splines of ANC levels in relation to crude HR (A) and adjusted HR(B) for the risk of MACCE.


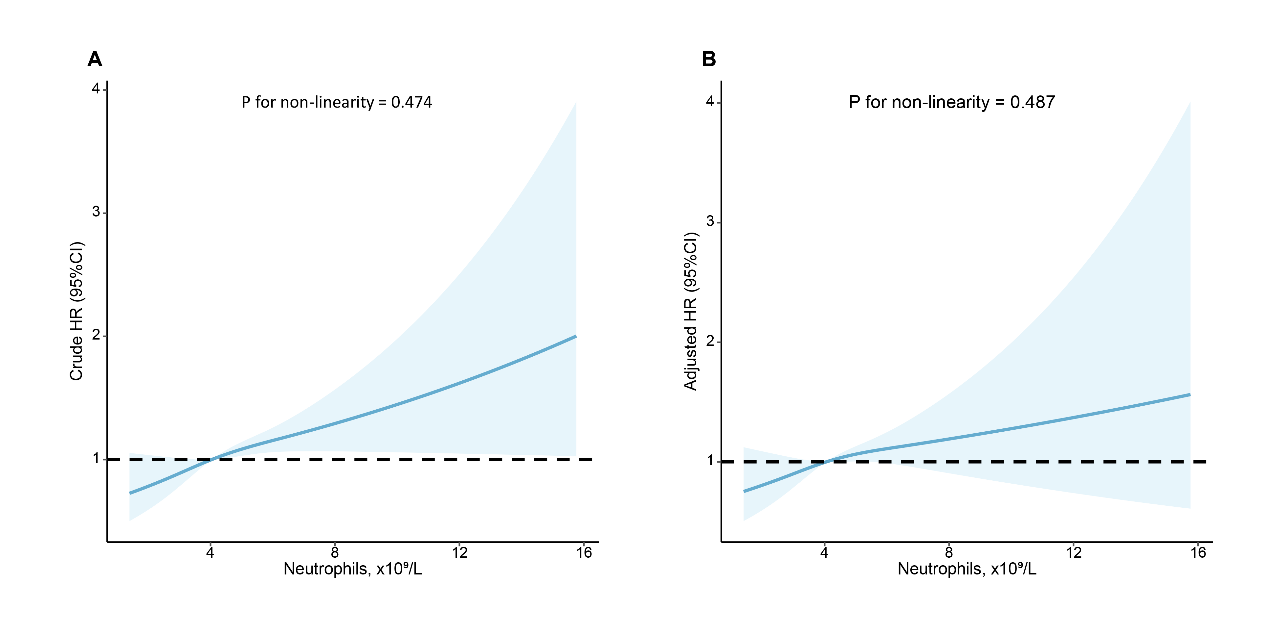


Model adjusted for age, male sex, BMI, hypertension, dyslipidemia, smoking history, previous MI, previous PCI/CABG, previous stroke, NSTE-ACS, hsCRP, eGFR, LVEF, LM/three-vessel disease, CTO lesions, number of treated vessels, and SYNTAX score.

Blue line with 95%CI shaded in light blue.

Abbreviations as in Table S1

**Fig. S2** Comparison of discrimination and reclassification ability of predictive models for MACCEs during follow-up.


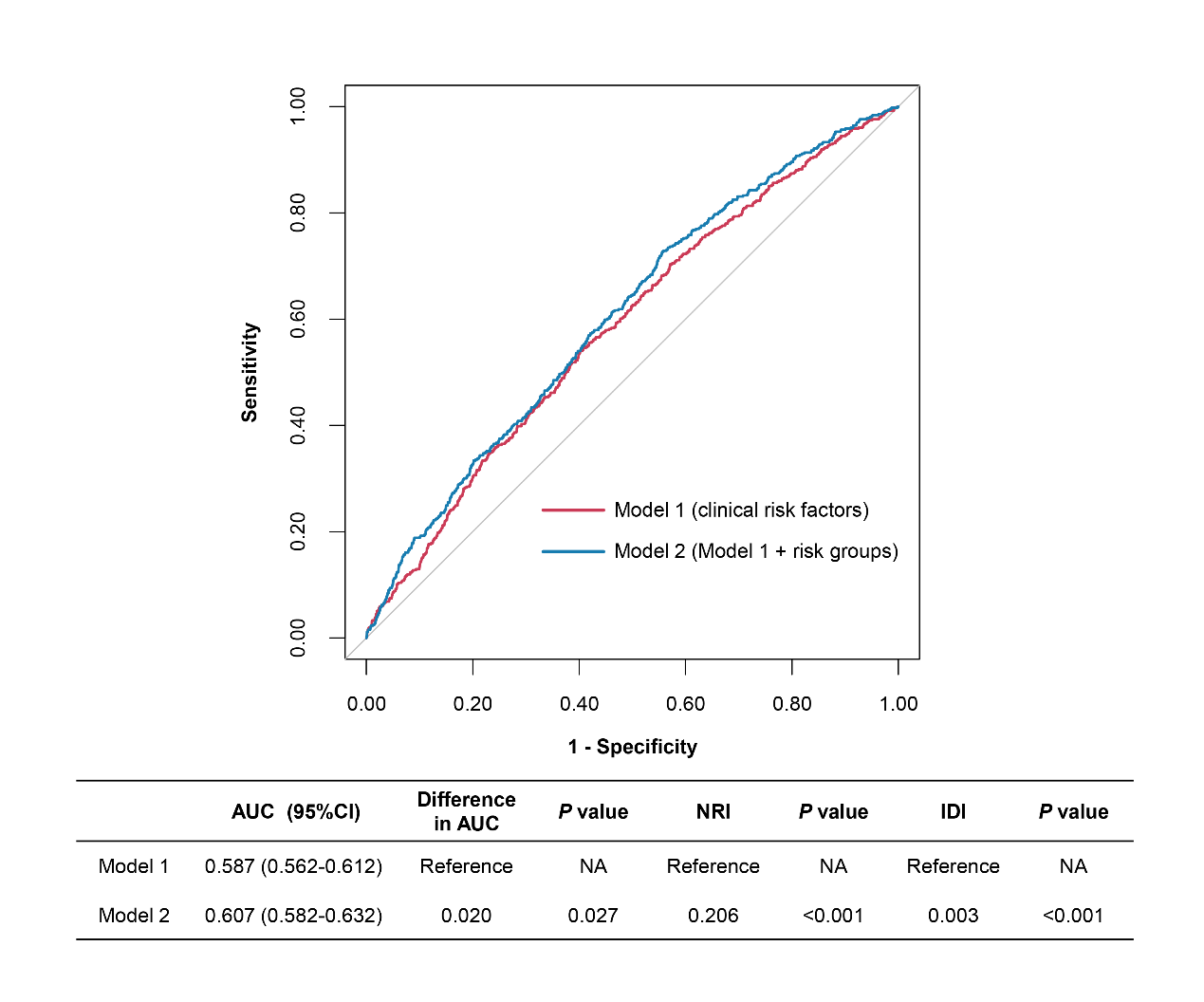


Discriminant functions to predict MACCEs during follow-up are presented. Model 1 included clinical risk factors alone, including age, male sex, hypertension, dyslipidemia, smoking history, previous stroke, previous MI, NSTE-ACS, LVEF, eGFR and SYNTAX score. Model 2 included clinical risk factors (model 1) plus risk groups with combination of ANC levels and glycemic metabolism status, which significantly increased discrimination and reclassification ability for predicting adverse events than model 1.

Abbreviations as in Table S1
